# Mechanisms of impairment of interferon production by SARS-CoV-2

**DOI:** 10.1042/BST20221037

**Published:** 2023-05-18

**Authors:** Huy-Dung Hoang, Parisa Naeli, Tommy Alain, Seyed Mehdi Jafarnejad

**Affiliations:** 1 Children's Hospital of Eastern Ontario Research Institute, Department of Biochemistry, Microbiology and Immunology, University of Ottawa, Ottawa, ON K1H 5B2, Canada; 2 Patrick G Johnston Centre for Cancer Research, Queen's University Belfast, Belfast, Northern Ireland BT9 7AE, U.K.

**Keywords:** antiviral immunity, coronavirus, innate immunity, interferons, NSP2, SARS-CoV-2

## Abstract

Interferons (IFNs) are crucial components of the cellular innate immune response to viral infections. The severe acute respiratory syndrome coronavirus 2 (SARS-CoV-2) has shown a remarkable capacity to suppress the host IFN production to benefit viral replication and spread. Thus far, of the 28 known virus-encoded proteins, 16 have been found to impair the host's innate immune system at various levels ranging from detection and signaling to transcriptional and post-transcriptional regulation of expression of the components of the cellular antiviral response. Additionally, there is evidence that the viral genome encodes non-protein-coding microRNA-like elements that could also target IFN-stimulated genes. In this brief review, we summarise the current state of knowledge regarding the factors and mechanisms by which SARS-CoV-2 impairs the production of IFNs and thereby dampens the host's innate antiviral immune response.

## SARS-CoV-2 infection and interferons

Coronavirus Disease 2019 (COVID-19) is caused by severe acute respiratory syndrome coronavirus 2 (SARS-CoV-2), a member of the Coronaviridae family and closely related to SARS-CoV and Middle East Respiratory Syndrome (MERS) coronaviruses [1]. Certain infected individuals with SARS-CoV-2 can experience severe symptoms that include thromboembolic complications, acute respiratory distress syndrome, tissue damage, and cardiac injury with potentially fatal consequences [2]. These severe COVID-19 cases are characterised by hyperinflammatory response resulting in multisystem organ failure and occurs more frequently with increased patient's age [3–6]. Localised and/or systemic inflammation, characterised by excessive production of certain cytokines such as Interleukin-6 (IL-6), IL-8, and TNFα has been linked to promoting such severe symptoms [7]. However, several lines of evidence indicate a highly protective role for another class of cytokines called interferons (IFNs), at least in the initial stages of SARS-CoV-2 infection. These include a host antiviral response to SARS-CoV-2 in nasal epithelial cells that is dominated by IFN signaling [8], repression of *in vitro* SARS-CoV-2 replication by type I IFN [9,10], and poor viral replication and less severe COVID-19 symptoms in individuals with a robust IFN-induced antiviral response in the early phase of infection [11,12]. Conversely, impaired or delayed production of type I IFN is associated with higher viral titers in blood and pernicious symptoms in late-stage patients [13,14]. Higher severity of SARS-CoV-2 infection is observed in individuals with various types of genetic deficiencies related to immunological defects affecting type I and III IFNs production [15–18] and Interferon-Stimulated Genes (ISGs) [19], or the presence of neutralising auto-antibodies against IFNs [18,20,21].

IFNs are important cytokines that exist in all jawed vertebrates. Their primordial roles are to confer protection against invading pathogens [22]. Human genome encodes three major classes of IFN: Type I, II, and III (Table 1). While type II IFNs are primarily produced by activated immune cells (e.g. NK and T-cells), types I and III can be produced by almost all cell types and are crucial as the first line of innate immune response to viral infections. In this review, we will highlight the important roles of type I and III IFNs in cellular responses to SARS-CoV-2 infection with a focus on the mechanisms by which SARS-CoV-2 impairs IFN production.

**Table 1:** Different types of Interferons and their upstream and downstream pathways

Type	Subtypes	Tissue specificity	Receptors	Downstream signaling pathway	Biological outcomes
I	α1, α2, α4, α5, α6, α7, α8, α10, α13, α14, α16, α17, α21, β, ε, κ, ω	-	IFNAR1/IFN AR2	JAK/STAT/IRF-9	Cellular and humoral immune responses
II	γ	NK, CD4^+^ & CD8^+^ T-cells	IFNGR1/IFN GR2	JAK/STAT JAK/STAT/IRF-9	Intracellular antiviral programs, polarisation and priming of macrophages to M1 phenotype
III	λ1, λ2, λ3, λ4	Epithelial cells	IFNLR1/IL10 Rβ	JAK/STAT	Localised immune response in epithelial tissues

## Type I and type III IFNs: the first line of defense against viral infection

Expression of type I and type III IFNs is regulated by a complex mechanism that is initiated by triggering of the pathogen-associated molecular pattern recognition receptors (PRRs). PRRs sense viral pathogen-associated molecular patterns (PAMPs) or host molecules associated with disturbance of homeostasis called danger-associated molecular patterns (DAMPs) [23]. There are five primary PRR families; Toll-like receptors (TLRs), retinoic acid-inducible gene I (RIG-I)-like receptors (RLRs), nucleotide-binding oligomerization domain (NOD)-like receptors (NLRs), C-type lectin receptors (CLRs), and absent from melanoma 2 (AIM2)-like receptors [23]. TLRs and RLRs provide the main mechanisms of detection of SARS-CoV-2 infection.

**Figure 1.**
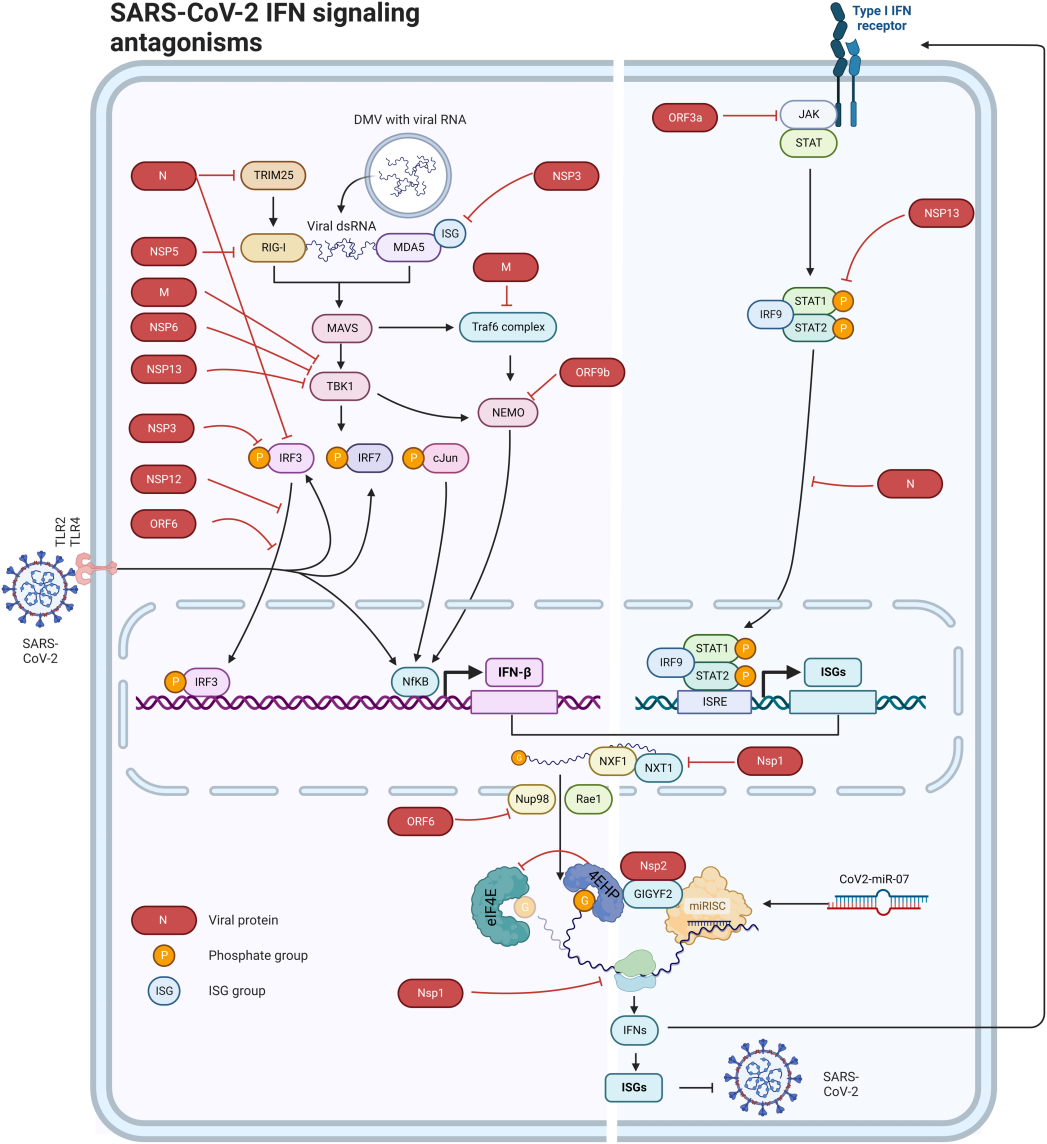
Schematic presentation of the mechanisms adopted by SARS-CoV-2 for disruption of the Interferon signaling pathway. Double-Membrane Vesicles (DMVs) derived from the endoplasmic reticulum membranes protect the dsRNA replication intermediates from recognition by TLRs and RLRs. NSP5 reduces the interaction between RIG-I and TRIM25 and cleaves a small 10-residue fragment at the N-terminal of RIG-I and thereby impairs the ability of RIG-I to activate IRF3. The N protein interacts with TRIM25 and RIG-I and suppresses RIG-I ubiquitination and activation. The M protein binds to and destabilises the RIG-I-MAVS–TBK1 interaction. NSP13 inhibits TBK1, while NSP6 reduces phosphorylation of IRF3. ORF9b inhibits the ubiquitination and activation of NEMO. NSP3 prevents MDA5 activation by removing the ISG15 groups, while blocking IRF3 activation by maintaining its ISGylation status. ORF6 and NSP12 block IRF3 nuclear translocation. NSP16 disrupts the host's mRNA splicing machinery by binding to the U1 and U2 snRNAs. NSP1 interacts with the host NXF1–NXT1 protein complex and prevents the export of cellular mRNAs. ORF6 impairs nuclear export by binding to KPNA2 and the nuclear pore complex proteins Nup98 and Rae1. NSP1 blocks mRNA translation via binding to the 40S ribosomal subunit at the mRNA entry tunnel. NSP14 in cooperation with NSP10 blocks mRNA translation by an unknown mechanism. NSP2 recruits and stabilises the GIGYF2/4EHP cap-dependent translational repressor complex to enhance the microRNA-mediated translational repression of target mRNAs including *Ifn1b* mRNA that encodes IFN-β. ORF3a promotes the ubiquitination and degradation of JAK2. NSP13 interacts with STAT1 and prevents its JAK1-mediated phosphorylation. The N protein impedes STAT1/2 phosphorylation and nuclear translocation.

TLRs are transmembrane proteins localised to the cell surface and in membrane bound organelles such as lysosomes, endosomes, or endolysosomes and they recognise distinct or overlapping PAMPs such as lipids, proteins, and nucleic acids [23]. Upon PAMPs and DAMPs recognition, TLRs generally transduce signals via two key adaptor molecules, MyD88 and TRIF, which initiate signal transduction pathways that result in the activation of IFN regulatory factors (IRFs), NF-κB, or MAP kinases and activation of transcription of genes that encode pro-inflammatory cytokines and type I IFNs (Figure 1). The first contact between SARS-CoV-2 and TLR sensors occurs at the cell surface, where SARS-CoV-2 envelope protein E and spike protein are recognised by TLR2 and TLR4, respectively [24,25]. Upon entering the cells, various SARS-CoV-2 derived molecules, including nucleocapsid (N), envelope (E), and spike (S) proteins, double-strand RNAs (dsRNAs), and single-strand RNAs (ssRNAs) can trigger various TLRs and activate the downstream signaling pathways [26–28]. Intracellular SARS-CoV-2-derived RNAs can also be sensed in the cytoplasm by RLRs (e.g. MDA5, LGP2, and RIG-I) that form a complex with the adaptor protein mitochondrial antiviral signaling (MAVS) [29,30]. This complex subsequently activates TNF receptor-associated factor 3 (TRAF3), TANK-binding kinase 1 (TBK1), and IκB kinase (IKK), which in turn phosphorylate and activate IRF3 and IRF7. Phosphorylated IRF3 and IRF7 form homo- or heterodimers that stimulate transcription of pro-inflammatory cytokines, type I IFNs and ISGs (Figure 1).

The resulting IFNs are subsequently secreted and stimulate downstream signaling through dedicated transmembrane receptors (Table 1) in autocrine and paracrine manners. While receptors for type I IFNs are widely expressed, the receptor for type III is restricted to specific cell types, namely macrophages, neutrophils, respiratory epithelial cells, conventional dendritic cells (DCs) and plasmacytoid dendritic cells (pDCs) [31]. Binding of type I and III IFNs to their respective receptors activates the JAK/STAT signaling pathway, which promotes the formation of the STAT1/2/IRF9 complex and stimulates the transcription of a large number of ISGs that mediate the establishment of an antiviral state (reviewed in [32]). A subset of ISGs can also be transcriptionally induced by other IRFs such as IRF3 [33]. Notably, due to the overlapping downstream signaling cascade for type I and III IFNs, many of their functional outcomes are similar. However, because of differences in the types of cells that express the dedicated receptors and the magnitude and kinetics of the response, distinct biological outcomes can result from activation of type I or type III IFNs. As such, type III IFNs are mainly found to serve as localised front-line defense in respiratory tissues, whereas type I IFNs are mostly produced by pDCs and can trigger both cellular and humoral immune responses to viruses via several pathways, which include inducing the expression of MHC II and co-stimulatory molecules, activating antigen presenting cells (APCs) and natural killer (NK) cells, and promoting the proliferation and differentiation of both CD8+ and CD4+ T-cells [34].

While a robust IFN-mediated antiviral response is required for combating viral infections, an exaggerated response upon viral recognition can lead to adverse consequences such as severe tissue damage [35]. Notably, aberrant expression of type I and III IFNs or dysregulated activation of ISGs has been frequently observed in the tissues or serum samples of patients with autoinflammatory and autoimmune disorders such as systemic lupus erythematosus, rheumatoid arthritis, Sjögren syndrome, and systemic sclerosis [36,37]. This highlights the imperative need for tight control of the expression of IFNs to maintain tissue homeostasis. However, such important and vital mechanisms are often ingeniously co-opted by viruses to dampen the antiviral immune response. SARS-CoV-2 has evolved several ways to target and control the IFN response.

## Mechanisms of evasion of IFN production by SARS-CoV-2

The SARS-CoV-2 virion contains four structural proteins: spike (S), envelope (E), membrane (M) and nucleocapsid (N), enveloping a 30 kb single-stranded positive-sense genomic mRNA. Once in the host cell, the genomic mRNA encodes two polyproteins from the open reading frames 1a (ORF1a) and ORF1b, which are subsequently cleaved by two viral proteases into sixteen non-structural proteins (NSP1-16). The viral genome also encodes the four structural (S, E, M and N) and up to ten accessory (3a, 3b, 3c, 6, 7a, 7b, 8, 9b, 9c, and 10) proteins.

The SARS-CoV-2 RNAs are recognised by TLRs, RIG-I, and MDA5 [29,38,39], which should result in the dramatic stimulation of innate immune response and in the transcriptional activation of IFN coding genes. However, overwhelming evidence suggests that SARS-CoV-2 infection induces IFNs and ISGs production only weakly [13,40,41]. Here, we summarise the various mechanisms adopted by SARS-CoV-2 to impair IFN production.

### Repression of activation of IFN coding genes

Masking the viral products that trigger the PRRs or blocking downstream signaling pathways are effective mechanisms to limit IFN production and evade the antiviral host response.

#### Blocking recognition by the host

SARS-CoV-2 employs several strategies to hide its genome and specifically target distinct antiviral proteins to limit the IFN response. SARS-CoV-2 protects its dsRNA replication intermediates from recognition by RLRs by replicating its ssRNA genome in double-membrane vesicles that are derived from the endoplasmic reticulum membranes [42]. The virus can also directly influence the post-translational modifications, protein–protein interactions, and activities of RLRs. The viral protein NSP5 is a highly conserved protease in Coronaviruses which is known to cleave a small 10-residue fragment at the N-terminal of RIG-I thereby impairing the ability of RIG-I to activate IRF3 [43]. NSP5 also reduces the interaction between RIG-I and TRIM25, which is required for the activation of RIG-I via its K63-linked ubiquitination by TRIM25 after recognising viral RNAs [44]. The N protein of SARS-CoV-2, despite being a structural protein, also interacts with TRIM25 and RIG-I, and suppresses RIG-I ubiquitination and activation [45–47].

#### Disrupting antiviral signaling pathways

Several viral proteins have been shown to block MAVS-induced signaling downstream of RLRs through distinct mechanisms. NSP13 interacts with and inhibits TBK1 phosphorylation at its S172 residue [48] either by blocking TBK1 autophosphorylation or the ability of IKKε to phosphorylate TBK1 [49]. NSP6 also binds to TBK1, leading to reduced downstream phosphorylation of IRF3, although without affecting TBK1 S172 phosphorylation [49]. Additionally, binding of the M protein inhibits the formation of the RIG-I/MAVS/TRAF3/TBK1 complex [50].

The interferon-stimulated gene 15 (ISG15) can conjugate to MDA5 at its caspase activation and recruitment domain through a process known as ISGylation [51]. MDA5 ISGylation induces its oligomerization and triggers activation of innate immunity. Through its papain-like protease (PLpro) activity, NSP3 antagonises MDA5 activation by removing the ISG15 groups [51]. ISGylation of several residues (Lys193, −360, and −366) on IRF3 by HERC5 results in sustained IRF3 activation by attenuating the interaction between Pin1 and IRF3 [52]. The SARS-CoV-2 protein NSP3 was shown to block IRF3 activation by de-ISGylating this important antiviral transcription factor [53]. The virus further inhibits IRF3 activity through ORF6, which blocks IRF3 nuclear translocation by binding to Karyopherin α2 (KPNA2), an importing factor for nuclear translocation of cargos [49]. The viral protein NSP12 also impairs IRF3 nuclear localisation, but not phosphorylation of this transcription factor [54]. Downstream of MAVS, the NFκB signaling pathway is targeted by ORF9b via its inhibition of ubiquitination of the modulator protein NEMO [55].

Interestingly, the JAK/STAT signaling that mediates the antiviral state of cells in an autocrine and paracrine manners downstream of type I IFN is also a target of SARS-CoV-2 disruption, perhaps affecting spread of the virus. The NSP13 protein interacts with STAT1 to prevent JAK1-mediated phosphorylation [56,57], while the N protein impedes STAT1/2 phosphorylation and nuclear translocation [58]. On the other hand, ORF3a promotes the ubiquitination and degradation of JAK2 via up-regulation of the suppressor of cytokine signaling 1 (SOCS1), a component of an E3 ubiquitin ligase complex that suppresses IFN signaling [59]. Through these mechanisms of blocking the recognition of the virus and the activation of the RIG-1/MAVS/TBK1 and the JAK/STAT pathways, SARS-CoV-2 acts to abolish the transcriptional activation of genes that encode type I and type III IFNs and ISGs.

### Post-transcriptional regulation of IFN production

Following transcription in the nucleus, mRNAs undergo multiple layers of post-transcriptional processes including splicing, nuclear export, translation, and finally degradation in the cytoplasm. Several lines of evidence have demonstrated that SARS-CoV-2 also employs various mechanisms to modulate virtually every step of post-transcriptional regulation of IFN production.

#### Interrupting mRNA splicing

Targeting the host splicing machinery is a strategy employed by some viruses to disarm the antiviral response [60,61]. In a screen for interaction between SARS-CoV-2 proteins and human RNAs, NSP16 was found to bind to the U1 and U2 snRNAs, important components of the spliceosome complex [62]. Overexpression of NSP16 disrupts global splicing, resulting in the defective splicing and intron retention in multiple IFN-responsive genes such as ISG15 and RIG-I, thereby impairing IFN signaling upon SARS-CoV-2 infection [62].

#### Blocking the mRNA nuclear export

Nuclear export of mRNAs is a crucial step in the mammalian gene expression process that is frequently targeted by different viruses to both thwart translation of host mRNAs and suppress the expression of IFN and other antiviral factors [63]. As SARS-CoV-2 completes its replication cycle within the cytoplasm, sabotaging the host nuclear export is a simple strategy to disrupt the cell's innate response without negatively impacting the expression of viral proteins. Genome-wide analysis of mRNA expression and stability upon SARS-CoV-2 infection revealed an accelerated rate of degradation of cellular mRNAs due to inhibition of nuclear mRNA export [64]. Single-molecule visualisation of host mRNAs during SARS-CoV-2 infection showed that in most infected cells SARS-CoV-2 inhibits the release of IFN-encoding mRNAs from their sites of transcription and triggers their decay in the nucleus [65]. NSP1 plays a key role in this process through interaction with the host NXF1–NXT1 protein complex, which is responsible for nuclear export of cellular mRNAs. NSP1 blocks binding of NXF1 to the export adaptors and docks at the nuclear pore complex to prevent the export of cellular mRNAs, including IFN-encoding mRNAs [66]. The viral protein encoded by ORF6 also impairs nuclear export and entraps host mRNAs including those of IFNs through binding to the nuclear pore complex (NPC) proteins Nup98 and Rae1 via its C-terminus [67–69].

#### Repressing mRNA translation

After being exported to the cytoplasm, cellular mRNAs are translated via the ribosomes with the aid of a large number of translation factors. As transcripts of antiviral genes ultimately need to be translated into protein, shutting down the host's mRNA translation is a strategy exploited by most viruses to allow redirecting the host's ribosomes toward the viral mRNAs and to impede the host antiviral response [70]. Thus, and perhaps not surprisingly, SARS-CoV-2 infection also leads to a reduction in global protein synthesis [62,64]. This effect is primarily attributed to NSP1, which inhibits translation by binding to the 40S small ribosomal subunit at the mRNA entry tunnel, rendering the ribosome inactive and leading to the degradation of the mRNA [64,71,72]. Concomitantly, the viral mRNA translation is maintained through a combination of two main mechanisms: 1. enhanced availability of ribosomes and translation factors due to degradation of cellular mRNAs [64], 2. elevated specific recruitment of ribosomes through a cap-proximal RNA element within the 5′ Un-Translated Region (5′ UTR) of the viral mRNAs that is devoid of guanosines and thus resists NSP1-mediated translation inhibition [73]. Besides NSP1, NSP14 also induces a near-complete shutdown in translation of cellular mRNAs, including those encoding IFN [49,74]. Although the exact mechanism by which NSP14 blocks mRNA translation and how it avoids repressing the translation of the viral mRNA is not clear, its exoribonuclease (ExoN) and N7-methyltransferase (N7-MTase) activities as well as its interaction with NSP10 are required for repression of mRNA translation [74].

In addition to shutting down general cap-dependent translation of cellular mRNAs, SARS-CoV-2 also employs a ‘targeted' mechanism of impairment of the host cell's IFN responses by recruiting the cap-binding and translational repressor eIF4E-homolog protein (4EHP). NSP2 recruits 4EHP through binding to the Grb10-interacting GYF (glycine, tyrosine, phenylalanine) protein 2 (GIGYF2). This interaction stabilises the GIGYF2/4EHP complex that specifically represses translation of *Ifnb1* mRNA, thereby impeding IFN-β production [75].

#### Targeting IFN-related transcripts by microRNAs and microRNA-like non-coding RNAs

4EHP and GIGYF2 mediate the translational repression of mRNAs targeted by microRNAs [69-71]. NSP2 enhances the microRNA-induced translational silencing through interaction with GIGYF2 and stabilisation of the GIGYF2/4EHP complex [76,77]. Once thought to be exclusively a host-related mechanism, microRNAs are also expressed by viruses to control both host and viral gene expression [78]. Studies using small RNA sequencing of SARS-CoV-2 infected cells identified virus-derived short RNA fragments that were later confirmed to be SARS-CoV-2 expressed microRNAs [79,80]. Such virus-derived microRNAs can be loaded into an Argonaute (Ago) protein, the key component of the microRNA-Induced Silencing Complex (miRISC) [80], indicating their ability to engage this machinery and repress the expression of the target RNAs. Notably, production of these virus-derived microRNAs is dependent on the cytoplasmic Dicer enzyme [80], yet independent of nuclear Drosha [78], suggesting that SARS-CoV-2 microRNAs might be expressed and matured in the cytoplasm, consistent with the subcellular location of virus replication. Computational predictions as well as empirical evidence demonstrate that these SARS-CoV-2 encoded microRNAs, most notably the miRNA CoV-miR-O7, could target several mRNAs that encode proteins with important roles in immune-regulatory processes both upstream and downstream of IFNs, such as IRF7, IRF9, STAT2, and ISGs (e.g. ISG15, MX1, and BATF2) [79,80,81,82]. Notably, multiple cellular microRNAs are also known to target the IFN mRNAs [83–85]. The potential role of NSP2 in exacerbating the repression of the target mRNAs of these host and virus-derived microRNAs in repression of the IFN production will be important to delineate.

## Perspectives

- The extent of the COVID-19 pandemic and its human and economic costs highlights the urgent need to fundamentally understand SARS-CoV-2 virus-host interactions. It is now clear that SARS-CoV-2 has acquired strong capacities to suppress the host IFN response, with an arsenal of proteins and RNA elements targeting virtually every step in the IFN production and signaling cascade.
- As new variants continue to emerge, defining the critical IFN-impairing functions of the distinct SARS-CoV-2 proteins, and monitoring how their important residues mutate over time to provide a viral advantage or induce more severe disease is important.
- Knowledge of the unique characteristics of the viral proteins that regulate expression of IFNs and innate immune responses could inform novel approaches in protein therapeutics against autoinflammatory and autoimmune diseases.

## Open Access

Open access for this article was enabled by the participation of Queen's University Belfast in an all-inclusive *Read & Publish* agreement with Portland Press and the Biochemical Society under a transformative agreement with JISC.

## Abbreviations

4EHP: eIF4E-homolog protein
ALRs: absent from melanoma 2 (AIM2)-like receptors
APCs: antigen presenting cells
CLRs: C-type lectin receptors
DAMPs: danger-associated molecular patterns
dsRNAs: double-strand RNAs
ExoN: exoribonuclease
GIGYF2: Grb10-interacting GYF (glycine, tyrosine, phenylalanine) protein 2
IFN: Interferon
IKK: IκB kinase
IL: Interleukin-6
IRFs: interferon regulatory factors
ISG: interferon-stimulated gene
ISGs: interferon-stimulated genes
JAK/STAT: Janus kinase (JAK)-signal transducer and activator of transcription (STAT)
MAVS: mitochondrial antiviral signaling
MHC: major histocompatibility complex
N7-MTase: N7-methyltransferase
NK: natural killer cells
NLRs: nucleotide-binding oligomerization domain (NOD)-like receptors
NSP: non-structural proteins
ORF: open reading frame
PAMPs: pathogen-associated molecular patterns
PRRs: pattern recognition receptors
RLRs: retinoic acid-inducible gene I (RIG-I)-like receptors
SARS-CoV-2: severe acute respiratory syndrome coronavirus 2
ssRNAs: single-strand RNAs
TBK1: TANK-binding kinase 1
TLRs: Toll-like receptors
TRAF3: TNF receptor-associated factor 3
